# cake_lpr: Verified Propagation Redundancy Checking in CakeML

**DOI:** 10.1007/978-3-030-72013-1_12

**Published:** 2021-02-26

**Authors:** Yong Kiam Tan, Marijn J. H. Heule, Magnus O. Myreen

**Affiliations:** 8 Eindhoven University of Technology, Eindhoven, The Netherlands; 9 Aalborg University, Aalborg East, Denmark; 10 Computer Science Department, Carnegie Mellon University, Pittsburgh, USA; 11 Chalmers University of Technology, Gothenburg, Sweden

**Keywords:** linear propagation redundancy, binary code extraction

## Abstract

Modern SAT solvers can emit independently checkable proof certificates to validate their results. The state-of-the-art proof system that allows for compact proof certificates is *propagation redundancy* (PR). However, the only existing method to validate proofs in this system with a formally verified tool requires a transformation to a weaker proof system, which can result in a significant blowup in the size of the proof and increased proof validation time. This paper describes the first approach to formally verify PR proofs on a succinct representation; we present (i) a new *Linear PR* (LPR) proof format, (ii) a tool to efficiently convert PR proofs into LPR format, and (iii) cake_lpr, a verified LPR proof checker developed in CakeML. The LPR format is backwards compatible with the existing LRAT format, but extends the latter with support for the addition of PR clauses. Moreover, cake_lpr is verified using CakeML ’s binary code extraction toolchain, which yields correctness guarantees for its machine code (binary) implementation. This further distinguishes our clausal proof checker from existing ones because unverified extraction and compilation tools are removed from its trusted computing base. We experimentally show that LPR provides efficiency gains over existing proof formats and that the strong correctness guarantees are obtained without significant sacrifice in the performance of the verified executable.

## 1 Introduction

Given a formula of propositional logic, the task of a SAT solver is to decide if there exists an assignment that satisfies the formula. Such a *satisfying assignment*, if found by a SAT solver, is easily verifiable by independent checkers and so one does not need to trust the inner workings of the solver. The situation with *unsatisfiable* formulas, i.e., where no satisfying assignment exists, is not as straightforward. Here, SAT solvers must produce an *unsatisfiability proof*. Ideally, the proof system (and proof format) for such proofs should be sufficiently expressive, allowing SAT solvers to efficiently produce proofs that correspond to the SAT solving techniques they use at runtime. At the same time, the resulting proofs ought to be efficiently checkable by independent and trustworthy tools.

The de facto standard proof system for propositional unsatisfiability proofs is known as Resolution Asymmetric Tautology (RAT) [24]. The associated DRAT format [36] combines clause addition based on RAT steps and clause deletion. Independent checking tools can validate proofs in the DRAT format; they have been used to check the results of the SAT competitions since 2014 [36] and in industry [15]. Enriching DRAT proofs with hints is the main technique for developing efficient verified proof checkers, e.g., existing verified checkers use the enriched proof formats LRAT [6] and GRAT [28].

A recently proposed proof system, called Propagation Redundancy (PR) [21], generalizes RAT. There exist short PR proofs without new variables for many problems that are hard for resolution, such as pigeonhole formulas, Tseitin problems, and mutilated chessboard problems [19]. Due to the absence of new variables it is easier to find PR proofs automatically [20], and it is considered unlikely that there exist short RAT proofs for these problems that do not introduce new variables nor reuse eliminated variables [21]. Such PR proofs can be checked directly [21], or they can first be transformed into DRAT proofs or even Extended Resolution proofs by introducing new variables [18,25]. In theory, the blowup is small, i.e., polynomial-sized. However, in practice, the transformed proofs can be significantly more expensive to validate compared to the original PR proofs [21].

A natural question arises: why should proof checkers be trusted to correctly check proofs if we do not likewise trust SAT solvers to correctly determine satisfiability? One answer is that proof checkers are much easier to implement so their code can be carefully audited. Another answer is that the algorithms underlying proof checkers have been *formally verified* in a proof assistant [6,15,28]. However, to get executable code for these verified checkers, some additional unverified steps are still required. Although unlikely, each of these steps can introduce bugs in the resulting executable: (1) the algorithms are extracted by unverified code generation tools into source code for a programming language; (2) unverified parsing, file I/O, and command-line interface code is added; (3) the combined code is then compiled by unverified compilers down to executable machine code.

The contributions of this paper are: (i) a new Linear PR (henceforth LPR) proof format that enriches PR proofs with hints and is backwards compatible with the LRAT format; (ii) a tool to efficiently enrich PR proofs with hints; and (iii) `cake_lpr`, an efficient verified LPR proof checker with correctness guarantees, including for steps (1)–(3) enumerated above. The `cake_lpr` tool is publicly available at https://github.com/tanyongkiam/cake_lpr and it was used to validate the unsatisfiability proofs in the 2020 SAT Competition because of its strong trust story combined with easy compilation and usage. Moreover, the stronger proof system could be supported in future competitions.

Section 3 shows how PR proofs can be enriched to obtain LPR proofs and presents the corresponding LPR proof checking algorithm (Contributions i & ii). Notably, existing LRAT proof checkers can be extended in a clean and minimal way to support LPR proofs. Section 4 explains the implementation of our checker in CakeML, as well as the correctness guarantees and high-level verification strategy behind the proofs (Contribution iii). Section 5 benchmarks our proof format

**Table 1.** A comparison of SAT proof checkers that have been verified in various proof assistants [6,15,28]. Green background (cells with +) indicates desirable properties, e.g., LPR is based on a stronger proof system than LRAT and GRAT, while red backgrounds (cells with ×) indicate less desirable properties. Yellow backgrounds (cells with −) are also undesirable but to a lesser extent.

| **Property** | ACL2 checker [15] | Coq checker [6] | GRATchk [28] | cake_lpr |
|---|---|---|---|---|
| Proof System (Section 3) | − LRAT | − LRAT | − GRAT | + LPR |
| Executable Code (Section 4) | − Directly Executed | × Unverified Extraction | × Unverified Extraction | + Binary Code Extraction |
| Checking Speed (Section 5) | + Fast | × Slow | + Very Fast | + Fast |

and proof checker against existing implementations. A summary comparison of the new proof checker against existing verified proof checkers is in Table 1.

## 2 Background

This section provides background on CakeML and its related tools. It also recalls the standard problem format and clausal proof systems used by SAT solvers.

### 2.1 HOL4 and CakeML

HOL4 is a proof assistant implementing classical higher-order logic [34]. CakeML is a programming language *deeply embedded* in HOL4, i.e., its abstract syntax is represented as a HOL datatype and its semantics is formalized within HOL4. Several tools for developing verified CakeML software are used in this work to fill the verification gaps in the correspondingly enumerated items in Section 1:

(1) Two tools are used to produce (or extract) verified CakeML source code:
   - the CakeML proof-producing translator [32] automatically synthesizes verified source code from pure algorithmic specifications;
   - the CakeML characteristic formula (CF) framework [14] provides a separation logic which can be used to manually verify (more efficient) imperative code for performance-critical parts of the proof checker.

(2) CakeML provides a foreign function interface (FFI) and a corresponding formal FFI model [10]. These are used to verify system call interactions, e.g., file I/O and command-line interfaces, under carefully specified assumptions.

(3) Most importantly, CakeML has a compiler that is *verified* [35] to preserve the semantics of source CakeML programs down to their compiled machine code implementations. Hence, all guarantees obtained from the preceding steps can be carried down to the level of machine code.

The combination of these tools enables *binary code extraction* [27] where verified machine code is extracted directly in HOL4. Several other CakeML-based programs have been verified using these tools, including: certificate checkers for floating-point error bounds [3] and vote counting [13], and an OpenTheory article checker [1]. Œuf provides a similar toolchain in the Coq proof assistant [31].

### 2.2 SAT Problems and Clausal Proofs

Fix a set of boolean *variables* $x_1, \ldots, x_n$, where the negation of variable $x_i$ is denoted $\overline{x}_i$, and the negation of $\overline{x}_i$ is identified with $x_i$. Variables and their negations are called *literals* and are denoted using $l$. The input for propositional SAT solvers is a formula $F$ in *conjunctive normal form* (CNF) over the set of variables $x_1, \ldots, x_n$. Here, CNF means that $F$ consists of an outer logical conjunction $F \equiv \bigwedge_{i=1}^{m} C_i$, where each *clause* $C_i$ is a disjunction over some of the literals $C_i \equiv l_{i1} \vee l_{i2}, \cdots \vee l_{ik}$. Formulas in CNF can be represented directly as sets of clauses and clauses as sets of literals. The empty clause is denoted $\bot$. An *assignment* $\alpha$ assigns boolean values to each variable; $\alpha$ can be *partial*, i.e., it only assigns values to some of the variables. Like formulas and clauses, a (partial) assignment can be represented as the set of literals assigned the boolean value true by that assignment. The negation of an assignment, denoted $\overline{\alpha}$, assigns the negation of all literals in $\alpha$. An assignment $\alpha$ *satisfies* a clause $C$ iff their set intersection is nonempty. Additionally, we define $C|_\alpha = \top$ if $\alpha$ satisfies $C$; otherwise, $C|_\alpha$ denotes the result of removing from $C$ all the literals falsified by $\alpha$, i.e., $C|_\alpha = C \setminus \overline{\alpha}$. For a formula $F$, we define $F|_\alpha = \{C|_\alpha \mid C \in F \text{ and } C|_\alpha \neq \top\}$. Intuitively, $F|_\alpha$ contains the remaining clauses in formula $F$ after committing to the partial assignment $\alpha$.

The task of a SAT solver is to determine whether $F$ is *satisfiable*, i.e., whether there exists a (possibly partial) assignment $\alpha$ such that $F|_\alpha$ is empty. Any satisfying assignment can be used as certificate of satisfiability. Formulas without a satisfying assignment are *unsatisfiable*. Certifying unsatisfiability is more difficult and typically uses a *clausal* proof system [21]. The idea behind these proof systems is briefly recalled next, using the key concept of clause redundancy.

**Definition 1.** *A clause $C$ is redundant with respect to formula $F$ iff $F \wedge C$ and $F$ are both satisfiable or both unsatisfiable, i.e., they are satisfiability equivalent.*

A clause $C$ that is redundant for $F$ can be added to $F$ without changing its satisfiability. Clausal proof systems work by successively adding redundant clauses to $F$ until the empty clause $\bot$ is added, as illustrated below:

$$F \overset{+\text{ redundant } C_1}{\Longrightarrow} F \wedge C_1 \overset{+\text{ redundant } C_2}{\Longrightarrow} F \wedge C_1 \wedge C_2 \overset{+\text{ redundant } C_3}{\Longrightarrow} \cdots \Longrightarrow F \wedge C_1 \wedge C_2 \wedge \cdots \wedge \bot$$

Satisfiability is preserved along each $\Longrightarrow$ step because of redundancy, e.g., satisfiability of $F$ implies satisfiability of $F \wedge C_1$. Since the final formula is unsatisfiable, the sequence of redundant clause addition steps $C_1, C_2, \ldots, \bot$ corresponds to a proof of unsatisfiability for $F$. Deciding clause redundancy is as hard as solving the SAT problem itself because $\bot$ is always redundant for unsatisfiable formulas. The difference between clausal proof systems is how the redundancy of a (proposed) redundant clause $C$ is efficiently certified at each proof step.

Many notions of redundancy are based on unit propagation. A *unit clause* is a clause with only one literal. The result of applying the *unit clause rule* to a formula $F$ is the formula $F|l$ where $(l)$ is a unit clause in $F$. The iterated application of the unit clause rule to a formula $F$ until no unit clauses are left is called *unit propagation.* If unit propagation on $F$ yields the empty clause $\bot$, denoted by $F \vdash_1 \bot$, we say that $F$ implies $\bot$ by unit propagation. The notion of *implied by unit propagation* is also used for regular clauses as follows: $F \vdash_1 C$ iff $F \wedge \neg C \vdash_1 \bot$ with $\neg C = \bigwedge_{l \in C} (\bar{l})$. Observe that $\neg C$ can be viewed as a partial assignment that assigns the literals $\bar{l}$, for $l \in C$, to true. For a formula $G$, $F \vdash_1 G$ iff $F \vdash_1 C$ for all $C \in G$. The main clausal proof system used in this paper is based on propagation redundant clauses, which are defined as follows.

**Definition 2.** *Let $F$ be a formula, $C$ a nonempty clause, and $\alpha$ the smallest assignment that falsifies $C$. Then, $C$ is* propagation redundant ($\mathsf{PR}$) *with respect to $F$ if there exists an assignment $\omega$ which satisfies $C$ and such that $F|_\alpha \vdash_1 F|_\omega$.*

Intuitively, a $\mathsf{PR}$ clause $C$ is redundant because any satisfying assignment for $F$ that does not already satisfy $C$ can be modified to a satisfying assignment for $F \wedge C$ by updating its literals assigned to true according to the (partial) witnessing assignment $\omega$ [21]. Propagation redundancy is efficiently checkable in polynomial time using the witnessing assignment and $\mathsf{PR}$ generalizes various other notions of clause redundancy, including the de facto standard Resolution Asymmetric Tautology ($\mathsf{RAT}$) proof system (see [21, Theorem 2]) that is able to compactly express all current techniques used in state-of-the-art SAT solvers [24].

In practice, clausal proof formats also contain deletion information to speed up proof validation. Hence, unsatisfiability proofs for formula $F$ are modeled as sequences $I_1, \ldots, I_n$ of *instructions* that either add or delete a clause. An *addition instruction* is a triple $\langle \mathsf{a}, C, \omega \rangle$, where $C$ is a clause and $\omega$ is a (possibly empty) *witnessing assignment*; a *deletion instruction* is a pair $\langle \mathsf{d}, C \rangle$ where $C$ is a clause. The sequence $I_1, \ldots, I_n$ gives rise to formulas $F_1, \ldots, F_n$ with $F_0 = F$ as follows, where $F_j$ is the *accumulated formula* up to the $j$-th instruction:

$$F_j = \begin{cases} F_{j-1} \cup \{C\} & \text{if } I_j \text{ is of the form } \langle \mathsf{a}, C, \omega \rangle \\ F_{j-1} \setminus \{C\} & \text{if } I_j \text{ is of the form } \langle \mathsf{d}, C \rangle \end{cases}$$

A $\mathsf{PR}$ proof of unsatisfiability is *valid* if the last instruction adds the empty clause $I_n = \langle \mathsf{a}, \bot, \emptyset \rangle$, and, for all addition instructions $I_j = \langle \mathsf{a}, C_j, \omega_j \rangle$, it holds that $C_j$ is $\mathsf{PR}$ with respect to $F_{j-1}$ using witness $\omega_j$. In case an empty witness is provided for $I_j$, then $F_{j-1} \vdash_1 C$ should hold.

## 3 Linear Propagation Redundancy

This section describes a new clausal proof format called LPR (short for Linear Propagation Redundancy). The format is designed to allow efficient validation

$$
\begin{array}{ll}
\langle \mathit{proof} \rangle & = \{\langle \mathit{line} \rangle\} \\
\langle \mathit{line} \rangle & = (\langle \mathit{lpr} \rangle \mid \langle \mathit{delete} \rangle), \text{“}\backslash n\text{”} \\
\langle \mathit{lpr} \rangle & = \langle \mathit{id} \rangle, \langle \mathit{clause} \rangle, \boldsymbol{\langle \mathit{witness} \rangle}, \text{“0”}, \langle \mathit{idlist} \rangle, \{\langle \mathit{reduced} \rangle\}, \text{“0”} \\
\langle \mathit{delete} \rangle & = \langle \mathit{id} \rangle, \text{“}d\text{”}, \langle \mathit{idlist} \rangle, \text{“0”} \\
\langle \mathit{reduced} \rangle & = \langle \mathit{neg} \rangle, \langle \mathit{idlist} \rangle \\
\langle \mathit{idlist} \rangle & = \{\langle \mathit{id} \rangle\} \\
\langle \mathit{id} \rangle & = \langle \mathit{pos} \rangle \\
\langle \mathit{lit} \rangle & = \langle \mathit{pos} \rangle \mid \langle \mathit{neg} \rangle \\
\langle \mathit{pos} \rangle & = \text{“1”} \mid \text{“2”} \mid \ldots \\
\langle \mathit{neg} \rangle & = \text{“}-\text{”}, \langle \mathit{pos} \rangle \\
\langle \mathit{clause} \rangle & = \{\langle \mathit{lit} \rangle\} \\
\boldsymbol{\langle \mathit{witness} \rangle} & \boldsymbol{= \{\langle \mathit{lit} \rangle\}}
\end{array}
$$

**Fig. 1.** The grammar for the LPR format. Additions compared to the LRAT grammar [6] are highlighted in bold.

of PR clauses using a (verified) proof checker. We also enhanced the `DPR-trim` tool[3] to efficiently add hints to PR proofs, thereby turning them into LPR proofs. Throughout the section, we emphasize how LPR can be viewed as a clean and minimal extension of the existing LRAT proof format, which thereby enables its straightforward implementation in existing LRAT tools.

The most commonly used proof format for SAT solvers is DRAT, which combines deletion with RAT redundancy [36]. DRAT proofs are easy for SAT solvers to emit and top-tier SAT solvers support it, but have some disadvantages for verified proof checking. In particular, checking whether a clause is RAT requires a significant amount of proof search to find the unit clauses necessary for showing the implied-by-unit-propagation property. This complicates verification of the proof checking algorithm and slows down the resulting verified proof checkers. The idea behind the Linear RAT (LRAT) [6, 15] and GRAT [28] formats is to include these unit clauses as hints so that verified proof checkers can follow the hints directly without the need for proof search. The LPR format lifts this idea to allow fast validation of the PR property.

An assignment $\omega$ *reduces* a clause $C$ if $C|_{\omega} \subset C$ and $C|_{\omega} \neq \top$. To check the PR property $F|_{\alpha} \vdash_1 F|_{\omega}$, it suffices to check, for each clause $C \in F$ reduced by $\omega$, that $F|_{\alpha} \vdash_1 C|_{\omega}$. Hence, in practice, a smaller $\omega$ yields a cheaper PR check. The LPR format extends the PR format by adding, for each clause that is reduced by the witness, a list of all unit clause hints required for showing the implied-by-unit-propagation property. Additionally, in order to point to clauses, the LPR format includes an index for each clause at the beginning of each line. The grammar of the LPR format is shown in Fig. 1.

Our extension to `DPR-trim` enriches input PR proofs by finding and adding all required unit clause hints. It also shrinks the witness $\omega$ where possible: every literal in $\omega \cap \alpha$ is removed as well as any literal in $\omega$ that is implied by unit propagation from $F|_{\alpha}$. The shrinking was shown to be correct [21], but has

[3] LPR hint addition is now part of the public GitHub version available at https://github.com/marijnheule/dpr-trim using the command-line option `-L`.

DIMACS file

```
p cnf 12 22
  1  2  3 0
  4  5  6 0
  7  8  9 0
 10 11 12 0
    -1 -4 0
    -2 -5 0
    -3 -6 0
    -1 -7 0
    -2 -8 0
    -3 -9 0
    ...
```

LPR proof file

```
23 -3 -10 -3 -10 1 12 0 -5 17  -8 20 -19  7 -22 10 0
24 -3 -11 -3 -11 2 12 0 -6 18  -9 21 -19  7 -22 10 0
25    -3                0                23 24   4 13 0
26 -6 -10 -6 -10 4 12 0 -5 11 -13  7 -14 20 -22 16 0
27 -6 -11 -6 -11 5 12 0 -6 12 -13  7 -15 21 -22 16 0
28    -6                0                26 27   4 19 0
29 -9 -10 -9 -10 7 12 0 -8 11 -13 10 -14 17 -19 16 0
30 -9 -11 -9 -11 8 12 0 -9 12 -13 10 -15 18 -19 16 0
31    -9                0                29 30   4 22 0
32    -2                0     6   9 28   31  2   3 14 0
33    -5                0     6  15 25   31  1   3  8 0
34                      0    25  28 32   33  1   2  5 0
```

**Fig. 2.** (Left) The first ten clauses of pigeonhole formula (4 pigeons, 3 holes) in the DIMACS format used by SAT solvers. (Right) The LPR refutation consisting of clause-witness pairs and unit clause hints. The first bold integer in each line is the clause index while other bold integers are the unit clause hints. Dropping the bold integers yields a proof in the PR format. Redundant spaces have been added to improve readability.

not been implemented so far. We observed that the witnesses in the PR proofs produced by SaDiCaL [20] can be substantially compressed using this method.

Fig. 2 (left) shows an example formula in the standard DIMACS problem format. The DIMACS format includes a header line starting with "p cnf " followed by the number of variables and the number of clauses. The non-comment lines (not starting with "c ") represent clauses, and they end with "0". Positive integers denote positive literals, while negative integers denote negative literals. Fig. 2 (right) shows a corresponding proof in LPR format. Deletion lines in LPR are formatted identically to LRAT [6] (not shown here). For clause addition lines, the LPR format only differs from LRAT in case the clause to be added has PR but not RAT redundancy. A clause addition line in LPR format consists of three parts. The first part is the first integer on the line, which denotes the index of the new clause. The second part consists of the clause and the witness; the first group of literals is the clause. The (potentially empty) witness starts from the second occurrence of the first literal of the clause until the first 0 that separates the unit clause hints. The second part exactly matches the PR proof format [21]. The third part (after the first 0) are the unit clause hints, which exactly matches the LRAT format [6].

The checking algorithm for LPR, shown in Fig. 3, overlaps significantly with that for LRAT (see [6, Algorithm 1]). The only differences are Steps 4 and 5.1. In Step 4, the witness is used (if present) instead of always using the first literal in $C_j$. In Step 5.1, clauses are skipped if they are satisfied by the witness. Notice that a clause can only be both reduced and satisfied by a witness if the witness consists of at least two literals, while in the LRAT format witnesses always consist of exactly one literal. Note also that the algorithm does not check whether $C_j\,|\omega = \top$, which is a requirement for PR. This omission is allowed because the first literal in $\omega$ in the LPR (and PR) format is the same as the first literal in $C_j$.

Input: CNF $F = \{C_i\}_{i \in \mathcal{I}}$ and line $\ell$ an LPR step.
Output: `YES` if parsed clause $C_j$ proved PR for $F$ by $\ell$,
`NO` otherwise.

1. parse $\ell$ as $\left[j, C_j, \boldsymbol{\omega_j}, 0, \widetilde{i^0}, \{-i^k, \widetilde{i^k}\}_{k=1}^{n}\right]$ instantiating variables with (vectors of) positive integers.
2. set $\alpha \leftarrow \neg C_j$
3. for $i \in \widetilde{i^0}$
   - 3.1. set $C'_i \leftarrow C_i \,|\, \alpha$
   - 3.2. if $C'_i = \bot$, return `YES`
   - 3.3. if $C'_i = \top$ or $|C'_i| \geq 2$, return `NO`
   - 3.4. set $\alpha \leftarrow \alpha \cup C'_i$
4. **if $\boldsymbol{\omega_j} \neq \emptyset$ then set $\boldsymbol{\omega} \leftarrow \boldsymbol{\omega_j}$ else** set $\omega \leftarrow (C_j)_1$ (if $C_j = \bot$, return `NO`)
5. for $i \in \mathcal{I}$
   - 5.1. if $C_i$ is **satisfied by $\boldsymbol{\omega}$** or is not reduced by $\omega$, skip to next iteration of Step 5.
   - 5.2. find $k$ such that $i^k = i$ (from $\ell$) (return `NO` if no such $k$ exists)
   - 5.3. if $C_i \,|\, (\alpha \setminus \overline{\omega}) = \top$, skip
   - 5.4. set $\alpha' \leftarrow \alpha \cup (\neg C_i \setminus \omega)$
   - 5.5. for $m \in \widetilde{i^k}$
     - 5.5.1. set $C'_m \leftarrow C_m \,|\, \alpha'$
     - 5.5.2. if $C'_m = \bot$, skip to next iteration of Step 5.
     - 5.5.3. if $C'_m = \top$ or $|C'_m| \geq 2$, return `NO`
     - 5.5.4. set $\alpha' \leftarrow \alpha' \cup C'_m$
   - 5.6. return `NO`
6. return `YES`

**Fig. 3.** Algorithm to check a single clause addition step in the LPR format. The bold parts show the additions compared to LRAT proof checking [6].

## 4 CakeML Proof Checking

This section explains the implementation and verification of `cake_lpr`, our verified CakeML LPR proof checker. Section 4.1 focuses on the high-level verification strategy which we used to reduce the verification task to mostly routine low-level proofs (the latter details are omitted). Section 4.2 highlights important verified performance optimizations used in the proof checker.

### 4.1 Verification Strategy

The development of `cake_lpr` proceeds in three refinement steps, where each step progressively produces a more concrete and performant implementation of the proof checker. These refinements are visualized in the three columns of Fig. 4.

Step 1 formalizes the definition of CNF formulas and their unsatisfiability, as well as the PR proof system described in Section 2.2. The inputs and outputs to

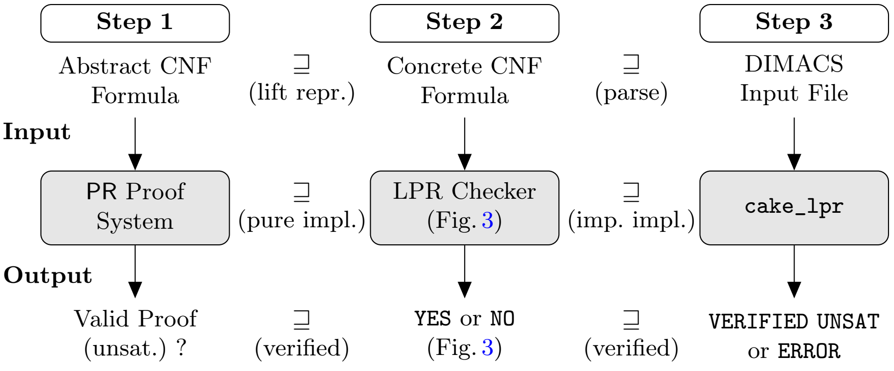

**Fig. 4.** The three step refinement used in the development of `cake_lpr`.

the proof system are abstract and not tied to any concrete representation at this step. For example, input variables are drawn from an arbitrary type $\alpha$, clauses and CNFs are represented using sets. The correctness of the PR proof system is proved in this step, i.e., we show that a valid PR proof implies unsatisfiability of the input CNF. The proof essentially follows [21, Theorem 1].

Step 2 implements a purely functional version of the LPR proof checking algorithm from Fig. 3. Here, the inputs and outputs are given concrete representations with computable datatypes, e.g., literals are integers (similar to DIMACS), clauses are lists of integers, and CNFs are lists of clauses. These concrete representations lift naturally to the abstract, set-based representation from Step 1. The output is a `YES` or `NO` answer according to the algorithm from Fig. 3. The correctness theorem for Step 2 shows that LPR proof checking correctly refines the PR proof system, i.e., if it outputs `YES`, then there exists a valid PR proof for the input (lifted) CNF; by Step 1, this implies that the CNF is unsatisfiable.[4]

Step 3 uses imperative features available in the CakeML source language, e.g., (byte) arrays and exceptions, to improve code performance; these optimizations are detailed further in Section 4.2. This step also adds user interface features like parsing and file I/O so that the input CNF formula is read (and parsed) from a file, and the results are printed on the standard output and error streams. The verification of this step uses CakeML's proof-producing translator [32] and characteristic formula framework [14] to prove the correctness of the source code implementation of `cake_lpr`; this code is subsequently compiled with the verified CakeML compiler. Composing the correctness theorem for source `cake_lpr` with CakeML's compiler correctness theorem yields the corresponding correctness theorem for the `cake_lpr` binary. The final correctness theorem is given in Appendix A. Briefly, it shows that if the `cake_lpr` executable prints the string "`s VERIFIED UNSAT\n`" to the standard output stream (in CakeML's FFI model [10]), then the input (parsed) DIMACS file is an unsatisfiable CNF.

[4] If the output is `NO`, the input CNF could still be unsatisfiable, but the input LPR proof is not valid according to the algorithm in Fig. 3.

### 4.2 Verified Optimizations

To minimize verification effort, CakeML's imperative features are only used for the most performance-critical steps of `cake_lpr`. Our design decisions are based on empirical observations about the LPR proof checking algorithm. These are explained below with reference to specific steps in the algorithm from Fig. 3.

**Array-based representations.** In practice, many LPR proof steps do not require the full strength of a PR (or RAT) clause. Hence, a large part of proof checking time is spent in the Step 3 loop of the algorithm and it is important to compute the main loop bottleneck, $C_i\,|\,\alpha$ in Step 3.1, as efficiently as possible. CakeML's native byte arrays are used to maintain a compact bitset-like representation of the assignment $\alpha$, so that $C_i\,|\,\alpha$ can be computed in one pass over $C_i$ with constant time bitset lookup for each literal in $C_i$.

For proof steps requiring the full strength of PR clauses, Step 5 loops over all undeleted clauses in the formula. Formulas are represented as an array of clauses[5] together with a lazily updated list that tracks all indices of the array containing undeleted clauses. This enables both constant-time lookup of clauses throughout the algorithm and fast iteration over the undeleted clauses for Step 5. Deletion in the index list is done in (amortized) constant time by removing a deleted index only when the index is looked up in Step 5.1. Additionally, for each literal, the smallest clause index where that literal occurs (if any) is lazily tracked in a lookup array; for a given witness $\omega$, all clauses occurring at indices below the index of any literal in $\overline{\omega}$ can be skipped in Step 5.1.

**Proof checking exceptions.** There are several steps in the proof checking algorithm that can fail (report `NO`) if the input proof is invalid, e.g., in Step 3.3. In a purely functional implementation, results are represented with an option: None indicating a failure and Some *res* indicating success with result *res*. While conceptually simple, this means that common case (successful) intermediate results are always boxed within an option and then immediately unboxed with pattern matching to be used again. In `cake_lpr`, failures instead raise exceptions which are directly handled at the top level. Thus, successful results can be passed directly, i.e., as *res*, without any boxing. Support for verifying the use of exceptions is a unique feature of CakeML's CF framework [14].

**Buffered I/O streams.** Proof files generated by SAT solvers can be large, e.g., ranging from 300 MB to 4 GB for the second benchmark suite in Section 5. These files are streamed into memory line by line because each proof step depends only on information contained in its corresponding line in the file. This streaming interaction is optimized using CakeML's verified buffered I/O library [29] which maintains an internal buffer of yet-to-be-read bytes from the read-only proof file to batch and minimize the number of expensive filesystem I/O calls.

[5] Deleted clauses are no longer referenced by the array and are automatically freed by CakeML's garbage collector.

## 5 Benchmarks

This section compares the verified CakeML LPR proof checker against other verified checkers on two benchmark suites and a RAT microbenchmark. The first suite is a collection of problems with PR proofs generated by the *satisfaction-driven clause learning* (SDCL) solver SaDiCaL [20], while the second suite consists of unsatisfiable problems from the SAT Race 2019 competition.[6] The RAT microbenchmark consists of proofs for large mutilated chessboards generated by a BDD-based SAT solver [5]. The CakeML checker is labeled cake_lpr (default 4GB heap and stack space), while other checkers used are labeled acl2-lrat (verified in ACL2 [15]), coq-lrat (verified in Coq [6]), and GRATchk (verified in Isabelle/HOL [28]) respectively. All experiments were run on identical nodes with Intel Xeon E5-2695 v3 CPUs (35M cache, 2.30GHz) and 128GB RAM. Configuration options specific to each benchmark suite are reported below.

### 5.1 SaDiCaL PR Benchmarks

The SaDiCaL solver produces PR proofs for hard SAT problems in its benchmark suite [20] and it is experimentally much faster than a plain DRAT-based CDCL solver on those problems [20, Section 7]. The PR proofs are directly checked by cake_lpr after conversion into LPR format with DPR-trim. For all other checkers, the PR proofs were first converted to DRAT format using pr2drat (as in the earlier approach [20]), and then into LRAT and GRAT formats using the DRAT-trim and GRATgen[7] tools respectively. All tools were ran with a timeout of 10000 seconds and all timings are reported in seconds (to one d.p.). Results are summarized in Tables 2 and 3.

All benchmarks were successfully solved by SaDiCaL except mchess19 which exceeded the time limit. For the remaining benchmarks, generating and checking LPR proofs required a comparable (1–2.5x) amount of time to solving the problems, except mchess, for which LPR generation and checking is much faster than solving (Table 2). Unsurprisingly, direct checking of LPR proofs is *much faster* than the circuitous route of converting into DRAT and then into either LRAT or GRAT (Table 3). Unlike LPR, checking PR proofs via the LRAT route is 5–60x slower than solving those problems; this is a significant drawback to using the route in practice for certifying solver results.

The backwards compatibility of cake_lpr is also shown in Table 3, where it is used to check the generated LRAT proofs. Among the LRAT checkers, acl2-lrat is fastest, followed by cake_lpr (LRAT checking), and coq-lrat. Although cake_lpr (LRAT checking) is on average 1.3x slower than acl2-lrat, it scales better on the mchess problems and is actually much faster than acl2-lrat on mchess18. We also observed that the GRAT toolchain (summing SaDiCaL, pr2drat, GRATgen and GRATchk times) is much slower than the LRAT toolchains

[6] The suites are available at http://fmv.jku.at/sadical/ and http://sat-race-2019.ciirc.cvut.cz/ respectively.

[7] GRATgen, the only tool that supports parallelism, was ran with 8 threads.

**Table 2.** Timings for PR benchmarks with conversion into LPR format. The "Total (LPR)" column sums the generation and checking times. The timing for `mchess19` is omitted because `SaDiCaL` timed out; timings for the Urquhart `U.-s3-*` benchmarks are omitted because they took a negligible amount of time (< 1.0s total).

| **Problem** | SaDiCaL | DPR-trim | cake_lpr (LPR) | Total (LPR) |
|---|---|---|---|---|
| hole20 | 1.0 | 0.5 | 0.7 | 2.2 |
| hole30 | 6.9 | 2.4 | 6.1 | 15.4 |
| hole40 | 31.3 | 10.0 | 25.1 | 66.3 |
| hole50 | 101.7 | 35.5 | 87.9 | 225.1 |
| mchess15 | 18.5 | 1.1 | 2.1 | 21.7 |
| mchess16 | 21.7 | 1.2 | 2.1 | 25.0 |
| mchess17 | 34.8 | 1.6 | 3.4 | 39.8 |
| mchess18 | 59.8 | 2.3 | 5.2 | 67.2 |

| **Problem** | SaDiCaL | DPR-trim | cake_lpr (LPR) | Total (LPR) |
|---|---|---|---|---|
| U.-s4-b1 | 0.7 | 0.6 | 0.3 | 1.6 |
| U.-s4-b2 | 0.3 | 0.4 | 0.2 | 0.8 |
| U.-s4-b3 | 0.4 | 0.4 | 0.2 | 1.0 |
| U.-s4-b4 | 0.3 | 0.5 | 0.3 | 1.1 |
| U.-s5-b1 | 2.5 | 0.9 | 1.3 | 4.7 |
| U.-s5-b2 | 1.2 | 0.6 | 0.7 | 2.4 |
| U.-s5-b3 | 3.2 | 1.5 | 2.0 | 6.8 |
| U.-s5-b4 | 5.5 | 1.5 | 3.2 | 10.1 |

**Table 3.** Timings for PR benchmarks, first converted to DRAT and subsequently converted into LRAT and GRAT formats. The "Total (LRAT)" and "Total (GRAT)" columns sum the fastest generation and checking times for the LRAT and GRAT formats respectively. The "Total (LPR)" column (in **bold**, fastest total time) is reproduced from Table 2 for ease of comparison. Fail(T) indicates a timeout. Timings for the `mchess19` and `U.-s3-*` benchmarks are omitted as in Table 2.

| **Prob.** | pr2drat | DRAT-trim | cake_lpr (LRAT) | acl2-lrat | coq-lrat | GRATgen | GRATchk | **Total (LPR)** | Total (LRAT) | Total (GRAT) |
|---|---|---|---|---|---|---|---|---|---|---|
| hole20 | 0.8 | 4.4 | 18.5 | 7.9 | 966.7 | 4.6 | 18.2 | **2.2** | 14.2 | 24.6 |
| hole30 | 6.8 | 61.4 | 180.4 | 105.9 | Fail(T) | 24.5 | 647.9 | **15.4** | 181.0 | 686.1 |
| hole40 | 32.4 | 460.0 | 1039.5 | 711.8 | Fail(T) | 101.3 | Fail(T) | **66.3** | 1235.5 | - |
| hole50 | 108.6 | 2663.0 | 4697.4 | 3292.2 | Fail(T) | 337.2 | Fail(T) | **225.1** | 6165.5 | - |
| mchess15 | 7.7 | 48.2 | 49.3 | 36.2 | Fail(T) | 48.4 | 2023.1 | **21.7** | 110.6 | 2097.7 |
| mchess16 | 9.0 | 62.0 | 59.8 | 53.2 | Fail(T) | 55.2 | 2903.8 | **25.0** | 145.9 | 2989.6 |
| mchess17 | 14.5 | 105 | 97.3 | 88.5 | Fail(T) | 86.1 | 7050.9 | **39.8** | 242.7 | 7186.3 |
| mchess18 | 25.1 | 195.0 | 152.7 | 296.8 | Fail(T) | 135.9 | Fail(T) | **67.2** | 432.5 | - |
| U.-s4-b1 | 0.5 | 2.5 | 3.6 | 3.3 | 135.7 | 3.6 | 44.8 | **1.6** | 7.0 | 49.7 |
| U.-s4-b2 | 0.2 | 0.8 | 1.4 | 1.0 | 23.2 | 1.7 | 8.2 | **0.8** | 2.3 | 10.4 |
| U.-s4-b3 | 0.3 | 1.3 | 2.0 | 1.5 | 49.2 | 2.4 | 16.2 | **1.0** | 3.5 | 19.3 |
| U.-s4-b4 | 0.3 | 1.1 | 1.8 | 1.4 | 38.3 | 2.0 | 10.3 | **1.1** | 3.1 | 12.9 |
| U.-s5-b1 | 4.2 | 13.6 | 16.7 | 12.5 | 3048.7 | 17.4 | 933.2 | **4.7** | 32.8 | 957.3 |
| U.-s5-b2 | 1.7 | 5.6 | 7.3 | 5.5 | 614.7 | 7.7 | 189.6 | **2.4** | 13.9 | 200.2 |
| U.-s5-b3 | 5.0 | 18.4 | 26.3 | 22.2 | 8750.5 | 21.1 | 2316.3 | **6.8** | 48.8 | 2345.6 |
| U.-s5-b4 | 11.3 | 34.2 | 36.9 | 30.1 | Fail(T) | 40.6 | Fail(T) | **10.1** | 81.0 | - |

(summing `SaDiCaL`, `pr2drat`, `DRAT-trim` and fastest LRAT checking times). This is in contrast to the SAT Race 2019 benchmarks below (Fig. 5), where we observed the opposite relationship. We believe that the difference in checking speed is due to the various checkers having different optimizations for checking the expensive RAT proof steps produced by conversion from PR proofs.

## 5.2 SAT Race 2019 Benchmarks

We further benchmarked the verified checkers on a suite of 117 unsatisfiable problems from the SAT Race 2019 competition. For all problems, DRAT proofs were generated using the state-of-the-art SAT solver `CaDiCaL` before conversion into the LRAT or GRAT formats. Notably, proofs generated by `CaDiCaL` on this

**Table 4.** A summary of the SAT Race 2019 benchmark results. The N/A row counts problems that timed out or failed in an earlier step of the respective toolchains.

| **Status** | CaDiCaL | DRAT-trim | acl2-lrat | cake_lpr | coq-lrat | GRATgen | GRATchk |
|---|---|---|---|---|---|---|---|
| Success | 102 | 97 | 96 | 97 | 36 | 100 | 100 |
| Timeout | 15 | 5 | 0 | 0 | 61 | 0 | 0 |
| Failure | 0 | 0 | 1 | 0 | 0 | 2 | 0 |
| N/A | 0 | 15 | 20 | 20 | 20 | 15 | 17 |

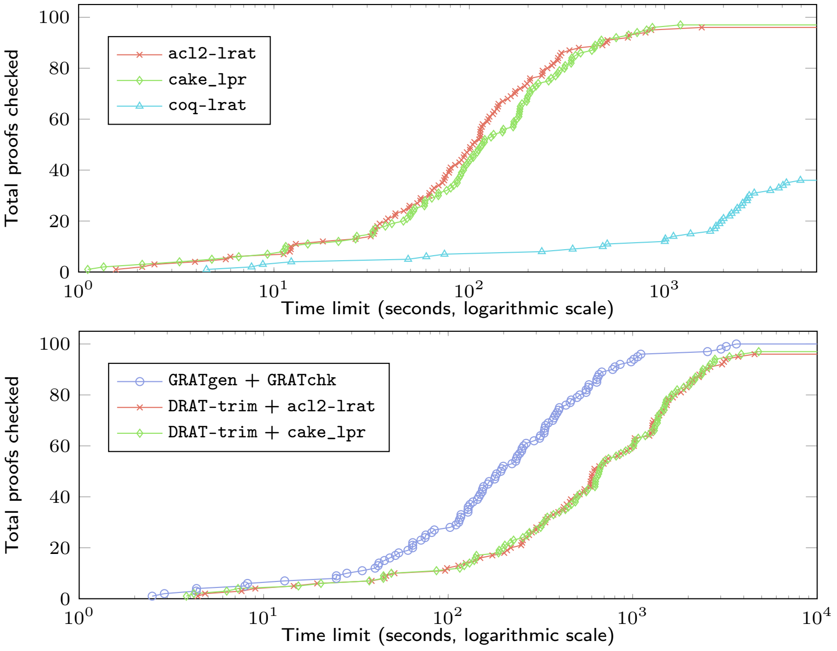

**Fig. 5.** (Top) Total SAT Race 2019 proofs checked within a given (per instance) time limit for the LRAT proof checkers. (Bottom) Total SAT Race 2019 proofs generated and checked within a given (per instance) time limit for the LRAT and GRAT toolchains.

suite rarely require RAT (or PR) steps, so the checkers are stress-tested on their implementation of file I/O, parsing, and Step 3.1 from Fig. 3; `cake_lpr` is the *only* tool with a formally verified implementation of the former two steps. All tools were ran with the SAT competition standard timeout of 5000 seconds.

A summary of the results is given in Table 4. All proofs generated by `CaDiCaL` were checked by at least one checker. The `acl2-lrat` checker fails with a parse error on one problem even though none of the other checkers reported such an error; `GRATgen` aborted on two problems for an unknown reason. Plots comparing LRAT proof checking time and overall proof generation and checking time (LRAT and GRAT) are shown in Fig. 5. From Fig. 5 (top), the relative order of LRAT checking speeds remains the same, where `cake_lpr` is on average 1.2x slower than `acl2-lrat`, although `cake_lpr` is faster on 28 bench-

**Table 5.** Timings for the RAT microbenchmark. The number of proof steps and file size of the proofs (in MB) are shown in the last two columns. Fail(T) indicates a timeout.

| **Problem** | pgbdd | lrat-check | cake_lpr | acl2-lrat | coq-lrat | LRAT Steps | File Size |
|---|---|---|---|---|---|---|---|
| mchess20 | 3.9 | 0.5 | 0.5 | 19.6 | 3405.2 | 125752 | 5.1 |
| mchess40 | 47.5 | 1.0 | 3.5 | 453.4 | Fail(T) | 769287 | 36 |
| mchess60 | 311.7 | 2.7 | 10.6 | 4885.2 | Fail(T) | 2300522 | 114 |
| mchess80 | 1164.1 | 4.8 | 22.6 | Fail(T) | Fail(T) | 5089457 | 259 |
| mchess100 | 3599.0 | 9.3 | 44.2 | Fail(T) | Fail(T) | 9506092 | 499 |

marks. From Fig. 5 (bottom), both LRAT toolchains are slower than the GRAT toolchain (average 3.5 times slower for `cake_lpr` and 3.4 times for `acl2-lrat`). Part of the speedup for GRAT comes from `GRATgen`, which is the only tool that can be ran in parallel (with 8 threads). This suggests that adding native support for GRAT-based input to `cake_lpr` could be a worthwhile future extension.

### 5.3 Mutilated Chessboard RAT Microbenchmarks

The final microbenchmark suite tests the LRAT checkers on large mutilated chessboard problem instances (up to 100 by 100) solved by `pgbdd`, a BDD-based SAT solver [5]. Unlike the previous two suites, LRAT proofs are emitted *directly* by the solver so additional `DRAT-trim` conversion is not needed. All tools were ran with a timeout of 10000 seconds and all timings are reported in seconds (to one d.p.). For additional scaling comparison, we also report results for `lrat-check`, an *unverified* LRAT proof checker implemented in C.

The results in Table 5 show the impact of `cake_lpr`'s RAT optimizations (Section 4.2). Notably, `cake_lpr` scales essentially *linearly* in the size of the proofs (up to $\approx$ 10 million proof steps). As a result, `cake_lpr` is significantly faster than `acl2-lrat` and `coq-lrat` on these RAT-heavy proofs and it comes within a 5x factor of the unverified `lrat-check` tool.

## 6 Related Work

*Verified Proof Checking.* There are several RAT-based verified proof checkers, in ACL2 [15], Coq [6], and Isabelle/HOL [28]. All three checkers are based on extensions of DRAT, which is itself an extension of the DRUP format [16]; the Coq checker is based on a predecessor for the GRIT [7] format. The ACL2 checker can be efficiently and *directly executed* (without extraction) using imperative primitives native to the ACL2 kernel [15]. However, the implementation of these features in ACL2 itself must be trusted to trust the proof checking results, hence the yellow background in Table 1. SMTCoq [2, 9] is another certificate-based checker for SAT and SMT problems in Coq. Its resolution-based proof certificates can be checked natively using native computation extensions of the Coq kernel.

*Applications.* SAT solving is a key technology underlying many software and hardware verification domains [4, 23]. Certifying SAT results adds a layer of trust and is clearly a worthwhile endeavor. Solver-aided mathematical results [17, 22, 26] are particularly interesting and challenging to certify because these often feature complicated SAT encodings, custom (hand-crafted) proof steps, and enormous resulting proofs [22]. Our cake_lpr checker can handle the latter two challenges effectively. For the first challenge, the SAT encoding of mathematical problems can also be verified within proof assistants. This was demonstrated for the Boolean Pythagorean Triples problem building on the Coq proof checker [8].

*Verified SAT Solving.* An alternative to proof checking is to verify the SAT solvers [11, 12, 30, 33]. This is a significant undertaking but it would allow the pipeline of generating and checking proofs to be entirely bypassed. Furthermore, such verification efforts can yield new insights about key invariants underlying SAT solving techniques compared to prior pen-and-paper presentations, e.g., the 2WL invariant [12]. However, the performance of verified SAT solvers are not yet competitive with modern (unverified) SAT solving technology [11, 12].

## 7 Conclusion

This work presents the new LPR proof format for verified checking of PR proofs. It demonstrates the feasibility of using binary code extraction to verify a performant LPR proof checker, cake_lpr, down to its machine code implementation.

Given the strength of the PR proof system, there is ongoing research into the design of *satisfaction-driven clause learning* techniques [20, 21] for SAT solvers based on PR clauses. Our proof checker opens up the possibility of using a verified checker to help check and debug the implementation of these new techniques. It also gives future SAT competitions the option of providing PR as the default (verified) proof system for participating solvers.

**Acknowledgments.** We thank Jasmin Blanchette and the anonymous reviewers for their helpful feedback on earlier drafts of this paper, Peter Lammich for help with GRATgen, and Stefan O'Rear for help with profiling CakeML programs.

The first author was supported by A*STAR, Singapore, the second author was supported by the National Science Foundation (NSF) under grant CCF-2010951, and the third author was supported by the Swedish Foundation for Strategic Research, Sweden. This work was also supported by NSF award number ACI-1445606 at the Pittsburgh Supercomputing Center (PSC).

## A Correctness Theorem for cake_lpr

The correctness theorem for cake_lpr verified in HOL4 is shown in Fig. 6. The assumptions (1) (in red) are routine for compiled CakeML programs that use its basis library. The first line assumes that the command-line *cl* and file system *fs* models are well-formed. The second line assumes that the compiled code is correctly placed into (code) memory according to CakeML's x64 machine model.

```
⊢ wfcl cl ∧ wfFS fs ∧ std_streams fs ∧ hasFreeFD fs ⇒                         }
  installed_x64 cake_lpr_code (basis_ffi cl fs) mc ms ⇒                        } (1)
  machine_sem mc (basis_ffi cl fs) ms ⊆                                        }
    extend_with_resource_limit                                                 } (2)
      { Terminate Success (cake_lpr_io_events cl fs) } ∧                       }
  ∃ out err.                                                                   }
    extract_fs fs (cake_lpr_io_events cl fs) =                                 }
      Some (add_stdout (add_stderr fs err) out) ∧                              }
    if out = «s VERIFIED UNSAT\n» then                                         } (3)
      (length cl = 3 ∨ length cl = 4) ∧ inFS_fname fs (el 1 cl) ∧              }
      ∃ mv fml.                                                                }
        parse_dimacs (all_lines fs (el 1 cl)) = Some (mv,fml) ∧                }
        unsatisfiable (interp fml)                                             }
    else if length cl = 2 ∧ inFS_fname fs (el 1 cl) then                       }
      case parse_dimacs (all_lines fs (el 1 cl)) of                            }
        None  ⇒  out = «»                                                      } (4)
      | Some (mv,fml)  ⇒  out = concat (print_dimacs fml)                      }
    else out = «»
```

**Fig. 6.** The end-to-end correctness theorem for the CakeML LPR proof checker.

The first guarantee (2) (in blue) is that the machine code implementation always terminates normally according to CakeML's x64 machine code semantics. In particular, the code never crashes and may emit some I/O events when run; however, it possibly terminates with an out-of-memory error (extend_with_resource_limit) when CakeML runs out of stack or heap space.

The main correctness guarantee for `cake_lpr` is (3) (in green) and (4) (in black). Briefly, (3) says that the only observable change to the filesystem after executing `cake_lpr` are strings printed on standard output *out* and standard error *err*. According to (3), if the string "`s VERIFIED UNSAT\n`" is printed onto standard output, then `cake_lpr` was provided with a file (in its first command-line argument), and the file parses in DIMACS format to a formula *fml* which is unsatisfiable. The remaining else case (4), says that the only other possibilities for standard output are either (i) a printed version of the parsed DIMACS file (if no LPR proof file is provided), or (ii) the empty string. All other error messages are printed onto standard error.

In addition, the DIMACS parser (parse_dimacs) is proved to be left inverse to the DIMACS printer (print_dimacs) in the following sense:

```
⊢ wf_fml fml ⇒
  ∃ mv fml′.
    parse_dimacs (print_dimacs fml) = Some (mv,fml′) ∧ interp fml = interp fml′
```

Briefly, this says that for any well-formed formula *fml*, printing that formula into DIMACS format then parsing it yields another formula *fml′* which is guaranteed to have the same interpretation according to the semantics of CNFs formalized in HOL4. All parsed formulas are well-formed (not shown here).
